# Spatial Associations of Long-term Exposure to Diesel Particulate Matter with Seasonal and Annual Mortality Due to COVID-19 in the Contiguous United States

**DOI:** 10.21203/rs.3.rs-1567636/v1

**Published:** 2022-07-15

**Authors:** Martine Mathieu, Joshua Gray, Jennifer Richmond-Bryant

**Affiliations:** North Carolina State University at Raleigh: North Carolina State University; North Carolina State University at Raleigh: North Carolina State University; US Environmental Protection Agency

**Keywords:** Diesel Particulate Matter, COVID-19 mortality, global models, geographically weighted regression, spatial pattern, seasonal effects

## Abstract

**Background:**

People with certain underlying respiratory and cardiovascular conditions might be at an increased risk for severe illness from COVID-19. Diesel Particulate Matter (DPM) exposure may affect the pulmonary and cardiovascular systems. The study aims to assess if DPM was spatially associated with COVID-19 mortality across three waves of the disease and throughout 2020.

**Methods:**

We tested an ordinary least square (OLS) model, then two global models, spatial lag model (SLM) and spatial error model (SEM), designed to explore spatial dependence, and a geographically weighted regression (GWR) model designed to explore local associations.

**Results:**

The GWR model found that associations between COVID-19 deaths and DPM concentrations may increase up to 57, 36, 43, and 58 deaths per 100,000 people in some US counties for every 1 μg/m^3^ increase in DPM concentration. Relative significant positive association are observed in New York, New Jersey, eastern Pennsylvania, and western Connecticut for the wave from January to May, and in southern Florida and southern Texas for June to September. The period from October to December exhibit a negative association in most parts of the US, which seems to have influenced the year-long relationship due to the large number of deaths during that wave of the disease.

**Conclusions:**

Our models provided a picture in which long-term DPM exposure may have influenced COVID-19 mortality during the early stages of the disease, but that influence appears to have waned over time as transmission patterns evolved.

## Background

In 2020, more than 20 million cases of coronavirus disease 2019 (COVID-19) were identified in the United States (U.S.), and more than 350,000 people died (Dong et al. 2020, John Hopkins University 2021). Many studies established the relationship between the prevalence of COVID-19 and underlying health conditions, social determinants of health, being of Black race, and environmental exposures. The most common underlying conditions reported to increase risk of severe illness from COVID-19 included obesity, hypertension, cardiovascular disease, type 2 diabetes, and chronic respiratory diseases, including asthma and chronic obstructive pulmonary disease (Clark et al. 2020, Nieman 2020). Incident COVID-19 deaths among people ages 65 years and older comprised at least 85% of all incident COVID-19 deaths in the U.S. on any day in 2020 (CDC 2021). In addition to age, socioeconomic status, access to healthcare, physical environment, and education have been identified as social determinants of COVID-19 hospitalization and mortality (Phillips et al. 2020, Mollalo et al. 2020). Several studies have observed a disproportionate share of COVID-19 incidence and mortality among predominantly Black U.S. communities, which may be partly attributable to social and economic inequalities and preexisting comorbidities (Yancy 2020, Reyes 2020, Phillips et al. 2020, Gayam et al. 2021, Peek et al. 2021). Yancy (2020) reported that the COVID-19 death rate in predominantly Black U.S. counties (6.3/100,000) was more than three times higher than in predominantly White counties in April 2020. Through July 21, 2020, Reyes (2020) reported a death rate twice as high among African Americans in the U.S. (97.9/100,000) compared with the White population, based on data from 45 states and the District of Columbia. Phillips et al. (2020) found that the increased risk of complications is related to the high prevalence of pre-existing comorbid conditions in part due to hereditary genetic predisposition.

The impact of particulate matter exposures on COVID-19 outcomes have also been evaluated, with some studies centered around diesel particulate matter (DPM). In an investigation of the role of long-term exposure (2000–2016) to air pollution during the first months of COVID-19, Wu et al. (2020) found that an increase of 1 μg/m^3^ in particulate matter with a nominal diameter of 2.5 μm (PM_2.5_) was associated with an 11% increase in the COVID-19 death rate for January 1-June 18, 2020. Bozack et al. (2022) performed a similar analysis to test associations of COVID-19 intensive care unit (ICU) admission and mortality with long-term concentrations of PM_2.5_, nitrogen dioxide, and black carbon for the period March 8-August 30, 2020 in New York City. They noted an association of ICU admission and mortality with long-term PM_2.5_ concentrations (collected December, 2018-December, 2019). Petroni et al. (2020) investigated the association of COVID-19 mortality with respiratory hazard index calculated across 3223 U.S. counties using emissions data for 2014 and COVID-19 data through May 13, 2020. They observed a 9% increase in COVID-19 mortality per unit increase in respiratory hazard index, which includes DPM. Their analyses with only DPM demonstrated an increased effect of 182% in the mortality rate ratio with a 0.5 μg/m^3^ increase in DPM concentration. Hendryx and Luo (2020) studied the association of long-term exposure to ozone (obtained from 2016), PM_2.5_ (obtained from 2016), and DPM (obtained from 2014) with COVID-19 prevalence and mortality through May 31, 2020. They showed an increase of 14.3 deaths per 100,000 U.S. residents for each DPM concentration increase of 1 μg/m^3^ in a single-pollutant model adjusted for demographic, health, smoking, and COVID-19 testing covariates. These findings collectively suggest that long-term PM exposure may predispose an individual to COVID-19 mortality. However, association of COVID-19 mortality with long-term DPM may change over time with the evolution of the coronavirus and changes in policies and personal behaviors. Our understanding of the effect of long-term DPM exposure on COVID-19 mortality during different waves of the disease and over the locations impacted by those waves remain unknown, hampering anticipation of disease hotspots.

DPM is composed of a complex mixture of black carbon and organic carbon. Studies have shown that 80–90% of particles emitted by diesel engines are less than 2.0 μm (Lee et al. 2015, Douglas et al. 2019), small enough to penetrate the alveoli (Ristovski et al. 2012). Long-term DPM exposure has been associated with adverse respiratory and cardiovascular effects (Pronk et al. 2009, Ristovski 2012, Douglas et al. 2019). Diesel engines power school buses, heavy-duty trucks, a variety of off-road heavy equipment, shipping, and commercial boating (Lee et al. 2015, Douglas et al. 2019). DPM emissions are higher in urban areas, where most of the global population lives (Ristovski 2012, Douglas et al. 2019). Likewise, greater DPM concentrations have been observed in socioeconomically disadvantaged communities (Douglas et al. 2019, Clougherty et al. 2014).

## Methods

Our study explores spatial associations between long term average concentrations of DPM, as a metric for past air pollution exposure, and COVID-19 mortality across each pandemic wave and throughout 2020 in the U.S. The objectives of the study are 1) to assess if living near DPM sources increased the risk of death from COVID-19, 2) to estimate how associations between mortality and long-term exposure to DPM may have changed over time with changes in the Coronavirus and in the population’s behavior, and 3) to test if models accounting for spatial autocorrelation improve model estimates. Data for air pollution, health, demographic, and social determinants of health were merged for this analysis, and global and local models were both applied to examine these relationships.

### Population Data

County-level number of COVID-19 deaths were obtained from the publicly-available Johns Hopkins Coronavirus Resource Center (Johns Hopkins University 2020) for the period January 1-December 31, 2020. Data for potential confounders associated with COVID-19 deaths, including access to health care, education, poverty, demographics, transportation, and occupation were obtained from the American Community Survey (ACS; U.S. Census Bureau 2020) and the County Health Rankings (CHR; Robert Wood Johnson Foundation 2020) (Table 1).

### Exposure Data

Long-term average DPM concentrations were obtained from the 2014 National Air Toxics Assessment (NATA) database, the most recently modeled concentrations of hazardous air pollutants and select other pollutants (U.S. Environmental Protection Agency (EPA) 2018). EPA used a hybrid model that coupled a Community Multiscale Air Quality (CMAQ) chemical transport model to the American Meterological Society/Environmental Protection Agency Regulatory Model (AERMOD) dispersion model to estimate NATA air pollutant concentrations at the census tract level through a multi-step process. CMAQ v5.2 was first run over a 12 km x 12 km grid based on DPM emissions inputs from the National Emissions Inventory (U.S. EPA 2018). Next, the AERMOD dispersion model was run for each source using the same inputs but with receptors distributed over census tract centroids. Finally, concentrations estimated by AERMOD along the census tract centroids were scaled by the ratio of the CMAQ concentration to the average of the AERMOD concentrations over that same grid cell. This formulation allows for more accurate representation of the chemistry and physics of the DPM than the AERMOD dispersion model can provide alone, while maintaining the finer census tract level spatial resolution of the dispersion model.

### Model Runs

We tested the association between COVID-19 mortality and long-term DPM concentrations across the contiguous United States for time periods coinciding with each COVID-19 wave in 2020: January 1-May 31, 2020, June 1-September 30, 2020, and October 1-December 31, 2020. We also ran the model for the entire year: January 1-December 31, 2020.

We used regression analysis to examine spatial non-stationarity in the relationship between COVID-19 and DPM while accounting for potentially confounding effects. This work is similar to spatial modeling approaches used by Sun et al. (2020) and Rahman et al. (2020). Sun et al. (2020) investigated different spatial regression models and compared them with an ordinary least squares (OLS) regression model to explain the transmission pattern of COVID-19. County-level race/ethnicity and socio-economic covariates were included in their models. We adapted their approach by focusing on associations of COVID-19 mortality with DPM and by investigating different time periods. Three global models, OLS, spatial lag model (SLM), and spatial error model (SEM), were run to produce a nationwide effect estimate. One local model, geographically weighted regression (GWR), produced effect estimates at the county scale. The R Statistical Software version 3.6.3 was used to run all code. We performed spatial regression modeling with the following libraries: *spdep*, *spgwr*, and *spatialreg*.

OLS models are designed to minimize the sum of squared differences between the true data and the prediction across the dataset (Goldberger, 1964). Mollalo et al. (2020) studied county-level variations of COVID-19 incidence in the U.S. From a list of 35 demographic, socioeconomic, topographic and environmental variables, they used a stepwise forward selection procedure and then checked for multicollinearity to determine the most significant predictors of COVID-19. Then, using the same selected explanatory variables, they tested their model using OLS and several spatial models including SEM, SLM, and GWR (described below). Accounting for spatial autocorrelation in their model improved performance over OLS. Karaye and Horney (2020) also compared OLS to spatial regression models to analyze the impact of social vulnerability on COVID-19 cases. Spatial autocorrelation of the residuals may compromise the validity of the OLS model and produce biased estimators (LeSage and Pace 2009, Loonis and De Bellefon 2018). The model assumptions of zero mean, independence, heteroscedasticity, and normal distribution are met for the case where OLS is a complete and correct model in which the variables capture all of the spatial variation without specifying spatial positions (DeAngelis and Yurek 2017, Schabenberger and Gotway 2017). Spatial autocorrelation in residuals may occur due to an omitted variable.

SLMs estimate an autocorrelation parameter (“spatial lag”) using a weighted average of the response variable across neighboring areas, testing if neighboring observations affect one another (LeSage and Fischer 2008, Sun et al. 2020). As the autocorrelation parameter approaches zero, the SLM approaches the OLS (LeSage and Fischer 2008). In SEMs, errors across neighboring areas are autocorrelated (“spatial error”) (Le Gallo et al. 2005). SEMs estimate the relationship between the residuals in a spatial region and those in adjacent regions (Sun et al. 2020). The spatial structure is in the residuals, meaning that some important predictors are omitted in the model (Chi and Zhu 2020).

SLM and SEM have only one spatial dependence parameter. The single-valued characteristic makes it impossible for global spatial models to reveal local spatial patterns (Chi and Zhu 2020, Fotheringham et al. 2003). Another limitation of global spatial models is that the model is dependent on the spatial weighting matrix (Chi and Zhu 2020 ). In contrast, GWR allows for local models to be fit to each observation using spatial distance as a weighting factor for the influence of all other points (Fotheringham et al. 2003). To determine local associations between COVID-19 cases in the U.S. and demographic, socio-economic, topographic and environmental parameters, Mollalo et al. (2020) examined two local models including GWR. The variables incorporated in the model are the same set used for OLS, SLM, and SEM. Similarly, Karaye and Horney (2020) compared GWR to OLS to understand the spatially varying effect in the relationship between social vulnerability and COVID-19 case counts. The main advantage of GWR as a local model is the ability to test for spatial variability among the effects of different variables in the model (Chi and Zhu 2020, LeSage and Pace 2009, Fotheringham et al. 2003). Another strength is that GWR has the same model structure as the OLS, which facilitates comparison between the two models (Fotheringham et al. 2003).

For our spatial autoregressive models, we estimated spatial relationships between regions based on contiguous boundaries shared between 2 or more counties, assuming that COVID-19 spread in a county is influenced by adjacent counties. For GWR, a cross validation function minimizes the root mean square prediction error that defines the weight matrix. We evaluated spatial autocorrelation among contiguous cells in the model residuals using Moran’s I (Moran 1950). Statistically significant Moran’s I indicates either correlation or anticorrelation among neighboring units. Additionally, we used Lagrange multiplier test statistics to understand whether the spatial lag or spatial error pattern is more important for interpreting the local results.

The level of urgency of the COVID-19 outbreak contributed to uncertain policy decisions and interventions in health in compressed timeframes coupled with the complex social, economic and political events of 2020 (Lancaster et al. 2020). Therefore, effects related to pandemic waves could have influenced the importance of specific variables during these different times of the year. Therefore, a set of different covariates have been integrated into the model for each time period. To determine which covariates to include in the regression models of COVID-19 mortality, we applied a stepwise selection algorithm for each season (Table 1). Then, the same covariates were incorporated in the best model for OLS, SLM, SEM, and GWR for each specific wave (Table 2), based on the following framework:

(1) COVID-19 deaths=DPM concentration+Confounder variables+error term

The confounder selection procedure was based on minimizing the Akaike information criterion (AIC) after controlling for multicollinearity. We used this same process for each of the three waves and throughout 2020 to find the most significant models for determining the nationwide and local associations between COVID-19 mortality and DPM concentration.

## Results

**Figure 1:**
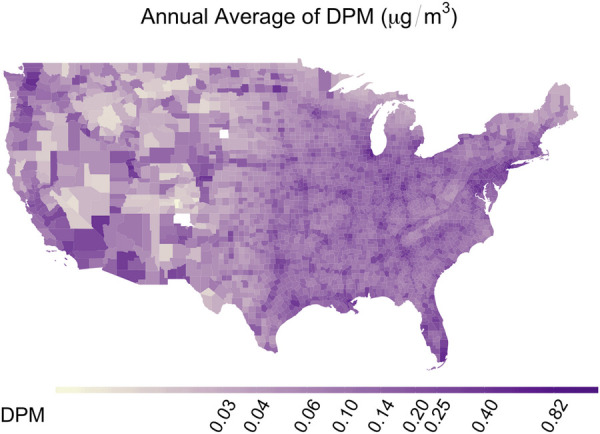
Spatial distribution of DPM concentration across contiguous U.S. counties (μg/m^3^).

County-level annual average DPM concentration varied from 0.0003 to 1.13 μg/m^3^ with a nationwide median of 0.095 μg/m^3^. Median regional concentrations for the Northeast, Southeast, West, and Mountain states were 0.117, 0.111, 0.079, and 0.037 μg/m^3^, respectively (Fig. 1). Elevated DPM concentration could be observed at specific points corresponding to cities.

**Figure 2:**
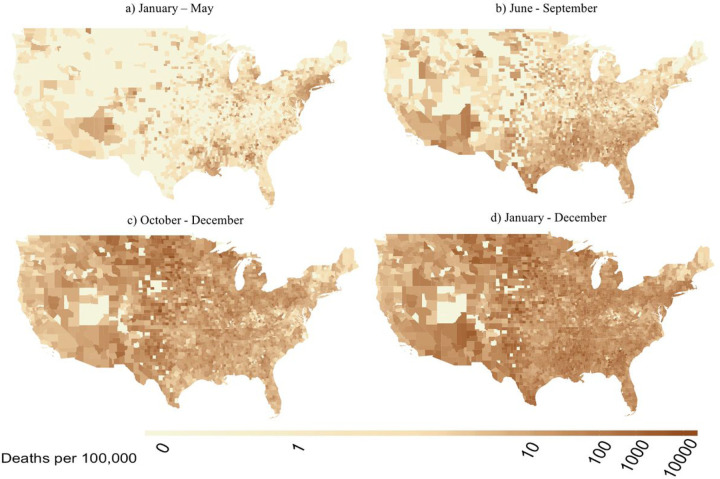
Spatial distribution of COVID-19 deaths for (a, top left) January-May, (b, top right) June-September, (c, bottom left) October-December, and (d, bottom right) all of 2020.

During the January May wave, the highest cumulative numbers of COVID-19 deaths were found in roughly the same regions as elevated DPM (Fig. 2a). As 2020 progressed, most counties experienced a higher mortality rate. The New York region exhibited lower cumulative deaths during the October-December wave of our study (Fig. 2c), with a mean of 98 deaths per 100,000 compared with the January-May wave, which had a mean of 280 deaths per 100,000 (Fig. 2a). As shown in Fig. 2a and 2b, cumulative deaths increased substantially from the first wave to the second wave in the Southeast region. In the West region, New Mexico, Arizona and California displayed the same pattern as the Southeast, with a significant increase during the second wave. For the September-December wave, COVID-19 deaths expanded across almost all of the US, exhibiting nearly the same pattern as for the all-year distribution (Fig. 2c and 2d).

At a global level, all models demonstrated a statistically significant association between long-term average DPM concentration and COVID-19 mortality for the first nine months of 2020, as represented by the January-to-May and June-to-September waves (Table 3). SLM and SEM produced slightly higher associations for the June-September wave. For the wave from October to December, none of the global models were found to produce positive associations or to be statistically significant. For the entire year, both the OLS and SLM produced positive associations, while the SEM produced a negative association.

OLS did not seem to be the most appropriate model to study spatial association between COVID-19 mortality and DPM. Smaller associations for the spatial autoregression models compared with OLS suggested that the OLS covariates were positively biased due to spatial autocorrelation. Both Moran’s I and visual inspection of the residuals maps (Figure S1) indicated spatial clusters of high values and of low values. The SLM and SEM models provided modest improvements in model fit, as indicated by slightly higher values of coefficient of determination (R^2^). Model fit testing indicates that the SLM provided a better fit than the SEM for the year-long data, based on the Lagrange multiplier test.

**Figure 3:**
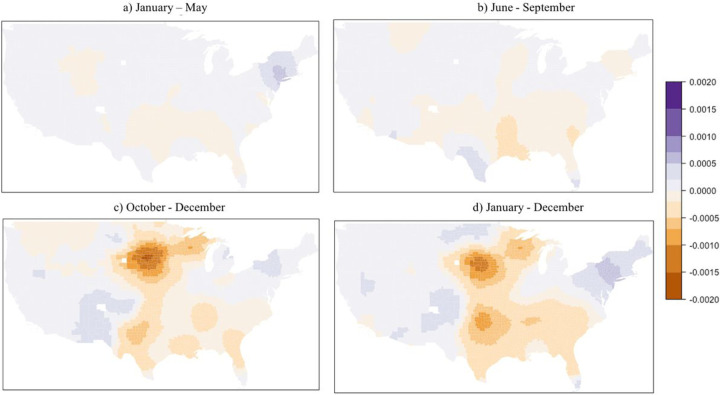
Map of associations between COVID-19 mortality and long-term DPM concentration for U.S. counties.

**Figure 4:**
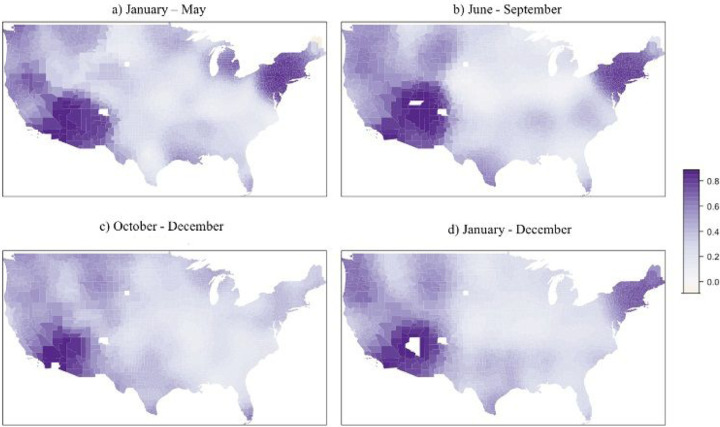
Spatial distribution of local R^2^ for the GWR model.

The local spatial differences estimated using the GWR model are presented as a range of values (Table 3). The mean COVID-19 mortality – DPM association for GWR is identical to that of the OLS, but overall R^2^ for GWR indicates improved performance over all global models. Spatial distribution of the DPM coefficients indicates changing conditions across the country during the three parts of the year (Fig. 3). During the January-May wave, associations were mostly positive across the U.S. (Fig. 3a), ranging from an increase of 57.19 deaths per 100,000 for every 1 μg/m^3^ increase in DPM concentration. During the June-September wave, about half of the contiguous US presented a positive association (Fig. 3b), while associations were more negative for the October-December wave (Fig. 3c). Year-round COVID-19 associations were similar to those for the October-December wave, likely due to the large number of cases during that timeframe. Local variations in R^2^ across the waves showed high (> 70%) values in the Northeast and Southwest during the January-May and June-September waves and in the year-long model. High R^2^ persisted into the October-December wave for the Southwest, albeit with a smaller area (Fig. 4). Low R^2^ (< 40%) were observed in the areas with greatest decrease in mortality with increasing DPM concentration, suggesting much greater uncertainty in those associations than in the positive ones seen in the New York area during the first wave. Moreover, COVID-19 mortality was only statistically significantly associated DPM concentration during the January-May wave.

Among all confounding covariates incorporated in the models, fraction Black race and fraction American Indian ethnicity were statistically significantly positive in all global models. In addition to these two covariates, Inactivity is significant in the June-September and October-December waves and in the year-long model, and the confounders Hispanic, Mining or Agriculture, Public Transportation, Time to Work, Income Inequality, and Population Density were significant at different time periods of the model.

## Discussion

Our study analyzed the spatial correlation between COVID-19 mortality and long-term DPM concentration as a surrogate for exposure across the continental United States during three waves of the COVID-19 pandemic during 2020. Our results suggested that long-term exposure to DPM may have been an important factor in COVID-19 mortality during the first two waves of the disease and that long-term DPM exposure may have been more highly influential during the January-May wave. Sidell et al. (2022) examined associations between air pollution exposure and COVID-19 incidence for monthly and annual averages of PM_2.5_, nitrogen dioxide (NO_2_), and ozone (O_3_) over four waves corresponding to those in our study plus January-February, 2021 for a Southern California cohort. They similarly observed that PM_2.5_ had a larger effect during the first wave and that the effect diminished over time. A spatial autocorrelation term was controlled for in these models, but Sidell et al. (2022) did not incorporate local methods. Differences in the outcome variable and the specific exposure also necessitates further examination of spatial and temporal patterns.

Our results indicate that the OLS does not account for the spatial associations of COVID-19 mortality with DPM concentrations. These results are similar to those of Sidell et al. (2022) and Mollalo et al. (2020), although their studies considered COVID-19 incidence rate rather than mortality. Mollalo et al. (2020) used OLS, SLM, SEM, and two versions of the GWR to model COVID-19 incidence and mortality for the time period of January 22-April 9, 2020 and found notable spatial associations of both COVID-19 incidence and mortality with several predictors. The study of Hendryx and Luo (2020), covering the January-May wave, revealed strong associations of COVID-19 prevalence and mortality with long-term DPM and PM_2.5_ concentrations. Their study estimated a coefficient of 14.3–18.7 deaths per 100,000 U.S. residents for each increase of 1 μg/m^3^ in DPM concentration. Inflation of the DPM effect shown in their results is possibly due to correlation between covariates and their mixed linear multiple regression model that does not account for spatial correlation. Stakhovych and Bijmolt (2007) emphasized that correlated spatial errors lead to bias and uncertainty in the OLS results. Moreover, LeSage and Fischer (2008) noted that spatial correlation in the OLS error terms is a sufficient motivation to employ spatial autoregression models for discerning spatial relationships between dependent and independent variables.

The spatial global models outperformed the OLS model in terms of model fit for all models except June-September. This improved performance may be related to spatial autocorrelation. A difference in coefficients and R^2^ among the OLS, SLM, and SEM models was not observed during the June-September wave, when the modeled relationships between COVID-19 mortality and long-term DPM concentrations lost statistical significance. Kim (2021) reported an inflated effect of spatial autocorrelation on OLS predictor coefficients, suggesting less spatial autocorrelation during the June-September wave consistent with Bini et al. (2009) and Smith and Lee (2011).

Among the modeling techniques analyzed for our study, GWR provided the best model fit, based on estimated global R^2^. Our results revealed where and when local long-term exposure to DPM may have been associated with COVID-19 mortality, consistent with results from both Karaye and Horney (2020) and Mollalo et al. (2020) regarding patterns of local prevalence and local mortality of the disease based on local R^2^. Some areas in the Northeast and West regions presenting a high R^2^ in our study align with Mollalo et al. (2020) for incidence rate.

As noted by Fotheringham (1998), our GWR results illustrates the need to account for local phenomena.

Socio-economic disparity could explain the non-stationary effect of DPM exposure on COVID-19 mortality, due to drastic differences between contiguous areas. Socially vulnerable communities, including minoritized racial groups, have seen spatially associated COVID-19 incidence (Karaye and Horney, 2020). This is consistent with the strong association we observed for the fraction Black confounder (Table 3). Moreover, Paolella et al. (2018) pointed out spatial associations among fine particulate matter concentration, health effects, and minoritized groups and found out that finer spatial resolution reveals substantially higher fine particulate matter concentrations in Black and Hispanic communities.

The differences among associations of COVID-19 mortality and DPM concentrations found by the SLM and SEM for the year-long time period, when SLM was demonstrated to be more significant by a Lagrange test, helped to illustrate that neighboring effects were more relevant in modeling the spatial relationship with COVID-19 deaths than unobserved latent variables contained in the error term. Counties near other counties with high COVID-19 incidence are likely to have higher incidence. Nonetheless, since the weighting matrix chosen for our study is based on spatial adjacency, the county size differences between the Eastern and Western U.S. may have affected the parameter estimates creating more uncertainty in the larger counties (Chi and Zhu 2020). Some variability in the association between COVID-19 and DPM exposure within those might have not been captured although DPM sources are more likely to be found in urban areas. However, since the SLM for the year-long time period was not statistically significant, other models should be considered when data are combined across multiple waves.

Several limitations of this study need to be acknowledged with respect to the input data. It is possible that, with more data and/or more time, the association would disappear. Exposure measurement error could bias the results (Villeneuve and Goldberg, 2020). Our spatial modeling approach is intended to account for spatial exposure measurement errors. However, errors from applying cross-sectional analyses persist. Although we studied different waves of the disease, our models were not truly longitudinal. Long-term exposure to DPM was estimated using concentrations from the 2014 NATA. This is the most recent nationwide prediction of DPM concentrations produced by the U.S. EPA and was also used in Hendryx and Luo (2020) and Petroni et al. (2020). The dataset likely includes higher DPM concentrations than for 2020 given fleet turnover, suggesting that the magnitude of the effects of DPM calculated by our study and these other studies were underestimated. Widely reported undercounting during the January-May wave would further contribute to this underestimation (Dubrow, 2021). The set of potential confounders employed in our models was chosen to evaluate the influence of factors other than DPM potentially associated with COVID-19 outcomes (Wu et al. 2020). However, it was impossible to represent all influential factors in the relationship between each wave of COVID-19 mortality and long-term DPM concentrations, so uncertainty in the potential for confounding existed (Wu et al. 2020, Hendryx and Luo 2020). Furthermore, the study was designed at county level. Spatial variation within counties was not captured and, the difference in county size could have caused uncertainty since the weighting matrix defined for our analyses on which SLM, SEM and GWR relied, was spatial adjacency. Therefore, associations at scales finer than county-level, including individual- and neighborhood-level associations, could not be inferred (Wu et al. 2020). Despite these limitations, our study included a rigorous analysis of spatial relationships for different time periods and tested a variety of potential confounders to minimize these limitations.

## Conclusions

Our study used spatial econometric models alongside a local GWR to assess spatial relationships between COVID-19 mortality waves and long-term DPM exposure. Our findings are consistent with prior studies showing a positive association between air pollution and COVID-19 mortality (Wu et al. 2020, Petroni et al. 2020), specifically for DPM (Hendryx and Luo 2020). Our study built on these previous findings by exploring associations of COVID-19 mortality with long-term DPM concentrations across waves of the pandemic. In doing so, our models provided a picture in which long-term DPM exposure may have influenced COVID-19 mortality during the early stages of the disease, as observed specifically for the periods of January-May and June-September 2020. Waning influence of DPM during October to December suggested that person-to-person disease transmission regardless of past exposures may have become more influential in the spread of COVID-19 and in mortality rates once the disease became widespread throughout the U.S. Further investigation might focus on factors associated with COVID-19 mortality during the October-December wave. Although COVID-19 data were available beyond this period, the introduction of vaccines during 2021 were likely to have been so influential that combination of the two years of data may produce misleading conclusions.

## Supplementary Material

Supplement 1

## Figures and Tables

**Table 1:** Potential confounders tested in the models.

**Race/ethnicity**	**Poverty and Wealth**
Fraction Black	Fraction homelessness
Fraction White	Fraction with a severe housing burden
Fraction Hispanic	Food-environment index
Fraction American Indian	Fraction Income inequality
Fraction Asian	Fraction Unemployment
Fraction Pacific Islander	Fraction Median income
**Transportation**	Fraction in poverty
Fraction who walks to work	Median home value
Fraction who takes public transportation to work	**Demographics**
Fraction who takes a bicycle or motorcycle to work	Population density
Fraction who drives a car to work	
Fraction average time to work	
Traffic volume	
**Age**	
Fraction over 65	
Median age	
**Occupation**	
Fraction in a service occupation	
Fraction in a manual occupation	
Fraction working in a mining or agricultural occupation	
Fraction working in a construction occupation	
Fraction working in a utilities occupation	
**Health**	
Fraction in poor health	
Fraction obese	
Fraction with diabetes	
Fraction reporting inactivity	
Fraction smoking	
**Access to Healthcare**	
Fraction uninsured	
Fraction population receiving health care	
Fraction hospitals per county	
Fraction hospital beds	
**Education**	
Fraction Incomplete school	

**Table 2:** Model framework for each wave modeled.

Wave Dates	Models
Jan 1-May 31, 2020	COVID-19 deaths = DPM concentration + Fraction Black + Fraction American Indian + Fraction who take public transportation to work + Fraction average time to work + Fraction uninsured + Fraction smoking + Fraction Income inequality + Population density (2)
Jun 1-Sep 30, 2020	COVID-19 deaths = DPM concentration + Fraction Black + Fraction Hispanic + Fraction American Indian + Fraction who take public transportation to work + Fraction reporting inactivity + Fraction Incomplete school + Population density (3)
Oct 1-Dec 31, 2020	COVID-19 deaths = DPM concentration + Fraction Black + Fraction American Indian + Fraction working in a mining or agricultural occupation + Fraction average time to work + Fraction reporting inactivity + Fraction obese + Fraction over 65 + Fraction homelessness (4)
Jan 1-Dec 31, 2020	COVID-19 deaths = DPM concentration + Fraction Black + Fraction Hispanic + Fraction American Indian + Fraction Pacific Islander + Fraction working in a mining or agricultural occupation + Fraction reporting inactivity + Fraction with a severe housing burden + Fraction Income inequality (5)

**Table 3:** Independent variables value per 100,000 people. Where cells are left blank, the forward stepwise variable selection process did not identify those variables for inclusion in the model. If a potential

Variables	January – May, 2020				June – September, 2020				October– December, 2020				January– December, 2020			
	OLS	SLM	SEM	GWR	OLS	SLM	SEM	GWR	OLS	SLM	SEM	GWR	OLS	SLM	SEM	GWR
DPM	3.05	1.9	2.05	−14.45–57.19	4.59	4.24	4.56	−30.63–36.49	−0.73	−1.12	−3.32	−157.98–43.21	5.56	1.5	−2.04	−116.1–58.0
Black	52.45	37.83	43.13	−906.15–540.33	109.9	86.73	105.28	−1148.6–414.02	−31.41	−18.35	−27.95	−3523.5–1267.7	124.3	69.51	110.85	−3368.2–1377.6
Hispanic	-	-	-	-	63.1	49.68	59.26	−246.24–280.38	-	-	-	-	77.91	54.8	95.82	−1031.3–8535.9
American Indian	35.4	30.31	27.8	−4001.9–639.59	59.69	57.98	60.75	−1349.6–8239.5	140.2	85.79	89.96	−2130.6–3225.9	167.5	122.82	115.26	−1652.2–5921.1
Pacific Islander	-	-	-	-	-	-	-	-	-	-	-	-	−4839	−3029	−2556	−24072–8391.4
Mining or Agriculture	-	-	-	-	-	-	-	-	403.4	192.43	177.57	-2076.3–2390.5	364.4	142.29	59.94	−2628.8–1654.4
Public Transportation	−539.9	−738.58	−853.25	−2742–3836.1	626.8	736.42	676.29	−10054–4309.5	-	-	-	-	-	-	-	-
Time to work	194.3	87.3	23.06	−1630.1–2133	-	-	-	-	−776	−347.01	−475.26	−5455.5–9276.3	-	-	-	-
Inactivity	-	-	-	-	113.3	96.5	90.52	−1447.5–4008.4	109.6	88.59	114.85	−677.69–816.65	178.9	136.29	177.95	−251.03–955.22
Uninsured	−54.69	−46.12	−37.53	−528.7–921.85	-	-	-	-	-	-	-	-	-	-	-	-
Smoking	−92.44	−82.97	−85.9	−1555.5–346.31	-	-	-	-	-	-	-	-	-	-	-	-
Obese	-	-	-	-	-	-	-	-	45.47	31.96	41.67	−536.1–742.75	-	-	-	-
Over 65	-	-	-	-	-	-	-	-	59.62	58.2	58.82	−810.75–891.23	-	-	-	-
Income inequality	3.81	3.55	2.85	−13.41–80.69	-	-	-	-	-	-	-	-	12.87	7.96	11.19	−26.16–91.04
Homelessness	-	-	-	-	-	-	-	-	−330.8	−184.44	−182.37	−2234.8–1384.1	-	-	-	-
Housing burden	-	-	-	-	-	-	-	-	-	-	-	-	−405	−228.9	−294	−1319.6–473.31
Incomplete school	-	-	-	-	1039	1009.8	1054.5	−3356.4–6255.9	-	-	-	-	-	-	-	-
Population density	0.013	0.013	0.016	−0.72 - −0.55	−0.014	−0.014	−0.015	−0.062–0.65	-	-	-	-	-	-	-	-
R^2^	0.37	0.42	0.45	0.65	0.41	0.44	0.44	0.59	0.17	0.33	0.32	0.48	0.15	0.32	0.32	0.42

## Abbreviations

ACS: American Community Survey
AERMOD: American Meterological Society/Environmental Protection Agency Regulatory Model
AIC: Akaike information criterion
CHR: County Health Rankings
COVID-19: Coronavirus disease 2019
DPM: Diesel particulate matter
EPA: Environmental Protection Agency
GWR: Geographically weighted regression
ICU: Intensive care unit
NATA: National Air Toxics Assessment
NO_2_: Nitrogen dioxide
OLS: Ordinary least squares
O_3_: Ozone
PM_2.5_: Particulate matter with a nominal diameter of 2.5 μm
R^2^: Coefficient of determination
SEM: Spatial error model
SLM: Spatial lag model
U.S.: United States
