# Supplementary material for: Spatial Associations of Long-term Exposure to Diesel Particulate Matter with Seasonal and Annual Mortality Due to COVID-19 in the Contiguous United States
Source: Res Sq. 2022 Jul 15:rs.3.rs-1567636. Preprint. [Version 1] doi: 10.21203/rs.3.rs-1567636/v1 (PMC9298138; doi:10.21203/rs.3.rs-1567636/v1)
Supplement: Supplement 1 [file NIHPPRS1567636v1-supplement-1.pdf]

Supplementary Files

This is a list of supplementary files associated with this preprint. Click to download.

- SupplementalInformationDPMmanuscript.docx
